# Cloning, expression, and purification of small subunit human ribosomal proteins SA/uS2, S2/uS5, S3/uS3, S7/eS7, S8/eS8, S12/eS12, S13/uS15, and S14/uS11 for *in vitro* characterization.

**DOI:** 10.17912/micropub.biology.002010

**Published:** 2026-07-09

**Authors:** Shay Nicholl, Madelyn Thompson, Athra Goshtasbi-Gowharrizi, Julia Streit, Jacob Martin, Daniel Outmezguine, Arman Charkhabi, Quira Zeidan

**Affiliations:** 1 First-Year Innovation & Research Experience (FIRE), Office of Undergraduate Research, University of Maryland, College Park, MD, USA

## Abstract

A recent paper describes an improved method for producing human ribosomal proteins in the soluble fraction of bacterial lysates rather than in inclusion bodies. We have extended this approach to clone, express, and purify additional proteins from the small ribosomal subunit for use in enzymatic and structural assays, as well as protein–protein interaction studies. Here, we show that an N-terminal 6×His-Trx tag enables the soluble expression and purification of human RPS3/uS3, RPS12/eS12, and RPS14/uS11 from *E. coli* cells. These results expand the repertoire of human ribosomal proteins available for individual characterization outside of the intact ribosomal complex.

**Figure 1. Cloning, expression, and purification of human ribosomal proteins using a 6xHis-Trx affinity and solubility tag:** A) Representative plasmid map for pNH-TrxT-RPS3/uS3 resulting from ligase-independent cloning of the human RPS3/uS3 coding sequence into the pNH-TrxT bacterial expression vector for inducible protein expression with N-terminal 6xHis-thioredoxin tag (the locations of the T7 promoter, ribosome binding site (RBS), and 6xHis-TrxA-RPS3/uS3 fusion ORF, among other features, are indicated). The figure was created using SnapGene 8.2 software. B) NuPAGE gel stained with Coomassie blue showing total protein bands from bacterial crude lysate after transformation with the indicated plasmids and small-scale protein expression tests at 18°C overnight after induction with IPTG. Lanes are: 1-2 “empty” pNH-TrxT, 3-4 pNH-TrxT-RPS3/uS3, 5-6 pNH-TrxT-RPS8/eS8, 7-8 pNH-TrxT-RPS13/uS15, 9-10 pNH-TrxT-RPS14/uS11. Samples from even-number lanes were induced with 0.5mM IPTG (+), whereas samples from odd-number lanes were grown concurrently through the experiment without induction (-). The arrows indicate the positions of the recombinant fusion protein bands at the predicted molecular weights, as listed in the table below the image. C) Same as B, for bacterial cells transformed with pNH-TrxT-RPSA/uS2 (left panel), pNH-TrxT-RPS2/uS5 (middle panel), or pNH-TrxT-RPS7/eS7 (right panel), exposed to 18°C overnight or 37°C for 2 hours, after induction (+). Uninduced samples were collected before IPTG addition (-). The arrowheads indicate the positions of the recombinant fusion protein bands at the predicted molecular weights, as listed in the table below the image. D) Bacterial growth curves from cells expressing 6xHis-Trx “empty” tag, 6xHis-Trx-RPS3/uS3, or 6xHis-Trx-RPS8/eS8 (upper panel) and 6xHis-Trx-RPS13/uS15 or 6xHis-Trx-RPS14/uS11 (lower panel) upon induction with 0.5 mM IPTG at 37°C, compared to the corresponding uninduced controls. E) Protein solubility and purification from sonicated bacterial lysates expressing 6xHis-Trx-tagged human ribosomal protein fusions after induction with 0.5 mM IPTG. The top panel shows a NuPAGE gel stained with Coomassie blue, which displays total protein bands from post-centrifugation samples (clear lysates) and Ni-NTA resin elutions. Western blots against the His tag, run simultaneously, are shown in the bottom panels. Lanes are: 1,6,10 pNH-TrxT-RPS3/uS3, 2,7,11 pNH-TrxT-RPS8/eS8, 3,8,12 pNH-TrxT-RPS13/uS15, 4,9,13 pNH-TrxT-RPS14/uS11, and 5 “empty” pNH-TrxT. The arrow indicates the position of the soluble RPS14/uS11 fusion protein at the predicted molecular weight (~30.3 kDa). F) Same as in E, but lysates were obtained from bead-disrupted cell pellets. Lanes are: 1 & 5 “empty” pNH-TrxT, 2 & 6 pNH-TrxT-RPS3/uS3, 3 & 7 pNH-TrxT-RPS13/uS15, and 4 & 8 pNH-TrxT-RPS14/uS11. The arrows indicate the position of the soluble RPS3/uS3 (~40.7 kDa), RPS14/uS11 (~30.3 kDa), and empty tag 6xHis-Trx (~15.7 kDa) fusion proteins at the corresponding predicted molecular weights. G) Purification of 6xHis-Trx-RPS12 from bacterial cells transformed with pNH-TrxT-RPS12/eS12 and induced with 0.5 mM IPTG overnight at 18ºC. Aliquots of 30 µL were collected from cleared lysate (CL, input), flowthrough (FT), three sequential washes (W1, W2, W3), and three elutions (E1, E2, E3), separated via NuPAGE and stained with Coomassie blue. The asterisk and arrow indicate the position of the recombinant fusion protein band at the predicted molecular weight of ~28.5 kDa in the cleared lysate and elutions, respectively.

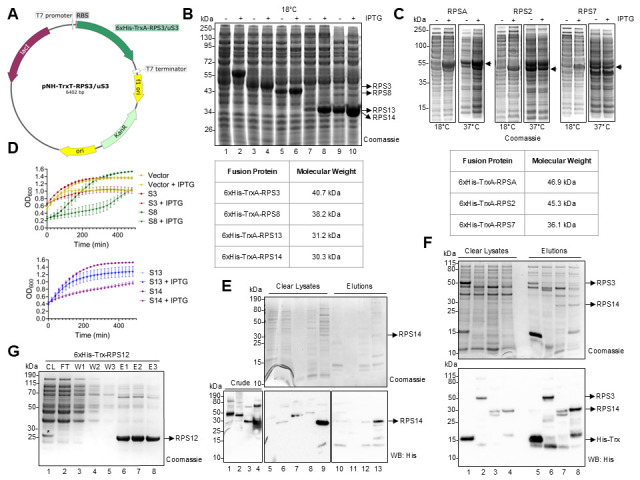



## Description

Ribosomal proteins are the structural scaffolds of the protein synthesis machinery (Palade, 1955). They are synthesized in the cytoplasm and, in eukaryotes, transported into the nucleus and nucleolus for assembly into ribosomal subunits (Warner et al., 1973). In humans, the large 60S subunit contains approximately 47 ribosomal proteins (and 5S, 5.8S, and 28S rRNAs), whereas the small 40S subunit harbors ~ 33 proteins (and 18S rRNA) (Kumar and Subramanian, 1975). Ribosomal proteins play a crucial role in ribosome biogenesis and translation and have numerous additional functions, ranging from activating tumor suppressor genes to an active role in the underlying mechanisms of certain genetic diseases (Kampen et al., 2020). 

Biochemically, ribosomal proteins have a high proportion of positively charged (basic) amino acids, such as lysine and arginine, whose electrostatic interactions with negatively charged rRNA promote subunit assembly and stabilize the mature ribosome (Lott et al., 2013). Newly synthesized ribosomal proteins also bind to dedicated chaperones that shield them from harmful interactions, facilitating transport to their appropriate location and preventing aggregation and premature degradation (Pillet et al., 2017). These *in vivo* interactions with assistive proteins and rRNA pose challenges when designing and implementing heterologous expression systems to produce individual ribosomal proteins, potentially requiring co-transformation and co-expression of multiple recombinant clones in the host organism. To overcome this challenge, a multi-tagging approach can generate recombinant fusion proteins with solubility-enhancing and affinity tags that boost soluble expression and facilitate purification of the target protein in bacteria (Bernier et al., 2018). This strategy has been recently used to successfully express and purify soluble human ribosomal proteins S10/eS10, S15/uS19, S18/uS13, and L11/uL5, fused to an N-terminal poly-histidine-thioredoxin (6xHis-Trx) tag in *Escherichia coli* (Correddu et al., 2019). This represents a significant improvement in the experimental methodology for producing and purifying human ribosomal proteins via a poly-histidine tag in bacteria, as previous efforts required protein recovery from insoluble inclusion bodies and refolding under harsh chemical conditions (Malygin et al., 2003).

In this study, we aimed to expand the repertoire of recombinant human ribosomal small subunit proteins expressed as soluble 6xHis-TrxA fusion constructs in *E. coli* , enabling rapid purification and subsequent *in vitro* characterization. To this end, we cloned the coding sequences of human ribosomal proteins SA/uS2, S2/uS5, S3/uS3, S7/eS7, S8/eS8, S12/eS12, S13/uS15, and S14/uS11 into the pNH-TrxT vector for inducible bacterial expression under control of the phage T7 promoter and a *lac* operator region (Savitsky et al., 2010). DNA sequencing results indicated successful insertion of the target ribosomal protein coding sequence in frame with an N-terminal poly-histidine-thioredoxin tag, followed by a specific recognition site for tobacco etch virus (TEV) protease cleavage for each construct ( Figure 1A ) (all the plasmids generated in this study are listed in Table 3 and deposited at Addgene). We transformed the recombinant plasmids into *E. coli* BL21 (DE3) competent cells and conducted small-scale protein expression experiments with 0.5 mM IPTG induction at 18°C overnight. For all the constructs tested (RPS3, RPS8, RPS13, RPS14), the presence of a high-intensity band at the expected molecular weight in the absence of IPTG ( Figure 1B, - IPTG lanes) represents a basal level of uninduced –leaky– gene expression commonly observed in recombinant elements derived from the pET system (Grossman et al., 1998). For the RPS3 and RPS8 fusion proteins, the addition of IPTG did not significantly change or only slightly increased protein abundance ( Figure 1B, lanes 3-6), whereas expression of the RPS13 and RPS14 constructs was enhanced considerably in the presence of the inducer ( Figure 1B, lanes 7-10). Derepression of the *lac* operator in the absence of IPTG may be mediated by a cellular response to nutrient shortage (Grossman et al., 1998) after overnight incubation at 18°C without media replenishment. To address this, we compared cultures of *E. coli* competent bacteria transformed with a subset of human ribosomal protein recombinant constructs (RPSA, RPS2, RPS7) that were grown overnight at 18 °C or for 2 hours at 37 °C after induction with IPTG. At the lower incubation temperature, these constructs exhibited negligible expression in the absence of the inducer after 8- 12 hours of incubation ( Figure 1C, 18°C panels). In contrast, incubation at a higher temperature for 2 hours (post-induction) showed recombinant protein bands at the expected molecular weight in both uninduced and induced samples, at comparable levels ( Figure 1C, 37°C panels). These results indicate that ensuring nutrient availability by limiting the incubation period (while increasing the temperature) does not prevent leaky expression of human ribosomal protein fusions constructed in the pET background. Using the pNH-TrxT system, five of the seven human ribosomal protein constructs tested showed increased expression following IPTG induction at 18 °C overnight. This suggests that lower incubation temperatures over extended periods may enhance the stability of these proteins when expressed in bacteria.

IPTG-induced exogenous protein overexpression in the pET/BL21 system triggers a bactericidal toxic effect, resulting in greater than 99.9% cell loss over time, independent of the recombinant protein's solubility (James et al., 2021). To test whether the abundance of exogenously produced human RP affects population viability in competent *E. coli* , we measured cell density by continuously monitoring optical density at 600 nm (OD _600_ ) over 8 hours at 37°C ( Figure 1D ). In cells transformed with empty vector, the addition of IPTG (at time zero) slightly suppressed population growth only after ~120 minutes, likely due to sustained accumulation of the 6xHis-Trx fusion ( Figure 1D, upper panel, yellow curves). The growth curve for cultures transformed with pNH-TrxT-RPS3/uS3 overlapped almost identically with that of the empty vector control under both uninduced and induced conditions ( Figure 1D, upper panel, red curves). In contrast, cells expressing 6His-Trx-RPS8 exhibited a marked slow-growth phenotype, evident at ~60 minutes upon addition of the inducer and maintained throughout the majority of the experiment ( Figure 1D, upper panel, green curves). These results suggest that, despite leaky expression and similar abundance ( Figure 1B ), the presence of the human RPS3/uS3 fusion protein does not impair bacterial growth dynamics, whereas the RPS8/eS8 fusion protein is harmful to cells. There was no difference in proliferation between cells expressing 6xHis-Trx-RPS13/uS15 and the uninduced control ( Figure 1D, lower panel, blue curves). In contrast, expression of 6His-Trx-RPS14/uS11 was mildly toxic throughout the experiment ( Figure 1D, lower panel, purple curves).

Predicting the toxicity of heterologous protein expression in the BL21(DE3) pET system has proven challenging because of gene-specific effects on the host cell's transcriptional response (Tan et al., 2020). To determine whether there was a correlation between the cytotoxic effect of human RP expression in bacteria and the protein solubility conferred by the 6xHis-Trx tag, we disrupted cell pellets from cells transformed with the indicated pNH-TrxT plasmid and induced with IPTG using sonication or bead homogenization. Lysates were centrifuged, and the cleared supernatants were subjected to protein denaturing electrophoresis, followed by Coomassie staining or Western blot analysis using an anti-His antibody. With the sonication method, a clear enrichment of the RPS14/uS11 fusion protein was observed in the soluble fraction, which was not seen for the other proteins, despite equivalent total protein loading (except lane 7) and initial abundance ( Figure 1E, Crude versus Clear Lysates, top and bottom panels). When bead homogenization was used, both the RPS14/uS11and RPS3/uS3 fusions were enriched in the soluble supernatant to a similar extent ( Figure 1F, top and bottom panels). Cleared lysates were used as the input sample for tagged-RP purification by affinity chromatography with Ni-NTA (Nickel-Nitrilotriacetic Acid) resin. Despite the presence of several high- and low-molecular-weight contaminants in the eluant, both disruption methods were suitable for purification of the RPS14/uS11 fusion ( Figure 1E- F). Additionally, the RPS3/uS3 fusion was also enriched in the eluted fraction from bead-homogenized samples, although the presence of low-molecular-weight species recognized by the His antibody indicates degradation products that retain the N-terminal tag ( Figure 1F ).

Although soluble expression of RPS3/uS3 and RPS14/uS11 as His-Trx fusions was successful, Ni-NTA purification did not yield a single predominant band in the elution fractions ( Figure 1E- F, upper panels). Purification efficiency in this system depends both on intrinsic protein properties, such as hydrophobicity and stability, and on experimental parameters, including IPTG concentration, codon optimization, and buffer composition (Rosano and Ceccarelli, 2014). Eukaryotic RPs exhibit intrinsic assembly behavior, including transient interactions with rRNA and biogenesis factors prior to stable incorporation into ribosomal subunits (Jakob et al., 2012). Notably, RPS3/uS3 and RPS14/uS11 participate primarily in late stages of small-subunit maturation (Ferreira-Cerca et al., 2005), whereas RPS12/eS12 associates with the earliest precursors of 40S biogenesis (Singh et al., 2021). To determine whether intrinsic biochemical properties contributed to the observed purification outcomes, we purified RPS12/eS12 from the soluble fraction of cells expressing pNH-TrxT-RPS12 ( Figure 1G ). Although RPS12/eS12 appeared as a moderately abundant band in the lysate ( Figure 1G, asterisk), the same purification protocol used for all proteins tested yielded highly enriched RPS12/eS12 with substantially fewer contaminants than observed for RPS3/uS3 and RPS14/uS11 ( Figure 1E- G, arrows). Because the contaminants present in the RPS12/eS12 preparation were detected at higher molecular weights (>30 kDa), the addition of a size-exclusion chromatography step could further improve purification outcomes. These findings suggest that the intrinsic biochemical properties of recombinant RPs are major determinants of purification efficiency in the His-Trx system.

Overall, our results demonstrate that the 6×His-Trx tag supports the soluble expression of human ribosomal proteins in *E. coli* , enabling the purification of RPS3/uS3, RPS12/eS12, and RPS14/uS11 by Ni-NTA affinity chromatography, albeit with varying degrees of purity. These observations indicate that additional optimization strategies may be required to obtain highly pure protein preparations. The data further suggest that both RP-specific biochemical properties and purification conditions influence purification outcomes. Future work will focus on refining affinity-purification protocols and integrating complementary approaches, such as size-exclusion chromatography following Ni-NTA purification, to generate highly pure, soluble recombinant RPs for in vitro, biophysical, and structural studies. More broadly, this work contributes to ongoing efforts to investigate the extraribosomal functions of human RPs and their potential roles in human disease.

## Methods

**Cloning of human ribosomal protein coding sequences into pNH-TrxT**

The method from Correddu et al. (2019) was adapted as follows. cDNA open reading frame (ORF) coding sequences of human RPs SA/uS2, S2/uS5, S3/uS3, S7/eS7, S8/eS8, S12/eS12, S13/uS15, and S14/uS11, cloned in pcDNA3.1+/C-(K)DYK, were purchased from GenScript (Table 1). RP DNA coding sequences were amplified by PCR (Phusion Plus DNA Polymerase, Thermo Scientific F630S) with primers containing 15-nt overlaps (Table 2) for cloning into the bacterial expression vector pNH-TrxT (Table 3). The resulting PCR fragments were separated by agarose gel electrophoresis, visualized using SYBR Safe DNA gel stain (Invitrogen S33102), and then extracted from the gel. The PCR-generated RP DNA coding sequences were inserted into PCR-linearized pNH-TrxT by ligase-independent cloning (In-Fusion Snap Assembly Master Mix, Takara Bio 638947) and transformation into Stellar competent cells (Takara Bio 636763). For each resulting recombinant plasmid, the RP DNA protein coding sequence replaced the SacB gene, located between nucleotides 426 and 2353, in the original pNH-TrxT vector. Recombinant pNH-TrxT-RP whole plasmid sequencing was performed by Plasmidsaurus using Oxford Nanopore Technology with custom analysis and annotation, followed by sequence alignment confirmation using Nucleotide Blast. All recombinant plasmids used in this study matched the expected sequence with 100% accuracy. Sequence-confirmed recombinant pNH-TrxT-RP plasmids (Table 3) were transformed into BL21 (DE3) competent cells (Thermo Scientific EC0114) and stored as bacterial glycerol stocks at -80°C. 

**Small-scale protein expression and protein analysis**

Glycerol stocks or fresh transformants of BL21 cells containing the pNH-TrxT-RP plasmid for each ribosomal protein, or an empty vector control, were used to inoculate 16-mL culture tubes containing 3 mL of LB medium supplemented with 50 µg/mL kanamycin. The cells were then grown overnight at 37°C in a shaking incubator. The next morning, the absorbance of the overnight cultures was measured using a visible light spectrophotometer (Thermo Scientific, Genesys 30). Dilutions were then inoculated into 3 mL of fresh medium at an optical density of 600 nm (OD _600_ ) of 0.1. Samples were incubated at 37°C in a shaking incubator for 2 to 2.5 hours, until an OD _600_ between 0.4 and 0.6 was reached, corresponding to the mid-log exponential phase. Pre-induction aliquots (1 mL) were taken from each sample, and isopropyl β-D-1-thiogalactopyranoside (IPTG) was added to the remaining culture to achieve a final concentration of 0.5 mM, initiating recombinant protein expression. Samples were immediately incubated at 37°C for 2 hours or at 18°C overnight with constant shaking at 250 rpm. Then, aliquots of 0.5-1 mL were collected post-induction. Pre- and post-induction samples were centrifuged at 3,000 rpm for 10 minutes. The supernatant was then removed, and the cell pellet was washed with 1 mL phosphate-buffered saline (PBS) by vortexing for 3-5 seconds. After centrifugation at 3,000 rpm for 10 minutes, the PBS was removed, and the cell pellets were stored at -20°C until further processed for protein analysis.

Pre- and post-induction bacterial cell pellets were mixed with 1X lithium dodecyl sulfate (LDS) sample buffer (Invitrogen NP0007) containing 5% (v/v) β-mercaptoethanol (BME), and incubated for 30 minutes at room temperature. The samples were then spun down at 13300 rpm to pellet the genomic DNA. The supernatants (crude extracts, 5-10 µL) were boiled at 100°C for 5 minutes, loaded onto a pre-cast 10% or 4-12% acrylamide gel (NuPAGE Bis-Tris Mini Protein Gels, Invitrogen), and electrophoresed at 130-150 V for 1-1.5 hours. Protein bands were visualized with Coomassie G-250 safe stain (Invitrogen LC6060), and gels were imaged using a ChemiDoc imaging system (Bio-Rad). 

**Growth curves**

Overnight starter cultures were prepared as described in the small-scale protein expression section. To generate bacterial growth curves using an absorbance microplate reader (Biotek ELx808), 190 µL of LB kanamycin media was dispensed into each well of a sterile 96-well plate, followed by the addition of 10 µL of each BL21 overnight culture to the corresponding wells. All dilutions were done using multi-channel pipettes and sterile reservoirs. After recording the initial OD _600_ for all samples, the microplate reader was set to monitor absorbance for each sample until an average OD _600_ of 0.4 was reached, corresponding to the mid-log exponential phase (elapsed time ~2.5 hours). At this point, plates were removed from the plate reader, and IPTG was added to a final concentration of 0.5 mM to the corresponding set of samples. The plate was returned to the microplate reader, and the protocol was resumed to measure cell density every 20 minutes for a total of 8 hours. Data were acquired using the Gen5 software (Agilent), and averages and standard error of the mean values were computed in Google Sheets. Graphs were plotted using GraphPad Prism 10.

**Purification of His-tagged fusion proteins and Western blot**

Overnight starter cultures were prepared as described above. The next morning, fresh cultures were inoculated at an OD _600_ of 0.1 in 10 mL of medium inside 50 mL conical tubes. Cultures were incubated and induced with IPTG overnight at 18°C, as described in the small-scale protein expression section. The next morning, samples were centrifuged at 5,000 rpm for 20 minutes at 4°C using a TX-400 swinging bucket rotor; the supernatant was then removed, and the cell pellet was washed with 1 mL of PBS. The cell pellet was then transferred to a 1.5 mL microcentrifuge tube and vortexed for 5-10 seconds. After another centrifugation step (5,000 rpm, 10 minutes), the PBS was removed, and the cell pellets were stored at -80°C until further processed. For solubility tests and affinity purification, pellets were disrupted using sonication or bead homogenization. For sonication, cells were resuspended in 1 mL of cell lysis buffer containing 50 mM Tris-HCl (pH 7.8), 500 mM NaCl, 10 mM imidazole, 10% (v/v) glycerol, supplemented with complete protease inhibitor (Roche) and 2 mM BME. Samples were lysed using a microtip sonicator (Branson 550) set to 45% amplitude, with up to 6x 30-second pulses alternating with 1-2 minutes on ice. For bead homogenization, cells were resuspended as above, transferred to 2 mL screw cap microtubes containing 2.8 mm ceramic beads (Omni International 19-628), and disrupted using a bullet blender (Next Advance BBX24B) for 2-4 cycles of 5 minutes each at maximum speed. Crude cell lysates were centrifuged at 15,000 x *g* for 15 minutes at 4°C, and the supernatants were collected for further analysis (clear lysates). Purification of His-tagged thioredoxin-ribosomal protein fusions was performed using HisPur Ni-NTA Resin (Thermo Scientific 88221) according to the manufacturer's batch method instructions, with a ratio of 500 µL lysate per 40 µL resin bed volume. Fractions were mixed with 2X LDS sample buffer, boiled for 5 minutes at 100°C, separated by gel electrophoresis, and visualized with Coomassie G-250 for loading control. Samples were transferred to a polyvinylidene difluoride (PVDF) membrane for Western blot analysis using a 6x-His tag monoclonal antibody (HIS.H8, Invitrogen, MA1-21315) diluted 1:2,000 in a blocking solution of 5% milk Tris-buffered saline with Tween-20 (TBS-T), followed by a goat anti-mouse IgG & IgM secondary antibody conjugated to HRP (Millipore Sigma AP130P) diluted 1:5,000 in blocking solution. Membranes were developed with Pierce ECL Western blotting substrate and imaged using a ChemiDoc imaging system.

## Reagents

Table 1. Human ribosomal protein cDNA ORFs used in this study

**Table:** 

**cDNA/ORF**	**Accession No.**	**GenScript Clone ID**	**Link to ORF Nucleotide Sequence**
RPSA (uS2)	NM_002295	OHu24758D	genscript.com/product/OHu24758D
RPS2 (uS5)	NM_002952	OHu03177D	genscript.com/product/OHu03177D
RPS3 (uS3)	NM_001005	OHu16953D	genscript.com/product/OHu16953D
RPS7 (eS7)	NM_001011	OHu18854D	genscript.com/product/OHu18854D
RPS8 (eS8)	NM_001012	OHu31095D	genscript.com/product/OHu31095D
RPS12 (eS12)	NM_001016	OHu30883	genscript.com/product/OHu30883
RPS13 (uS15)	NM_001017	OHu13042D	genscript.com/product/OHu13042D
RPS14 (uS11)	NM_001025070	OHu10378D	genscript.com/product/OHu10378D

Table 2. Custom primers used in this study (obtained from Integrated DNA Technology)

**Table:** 

**Primer Name**	**Primer Sequence (5′ to 3′)**	**Used for**
Vector.FOR	cagtaaaggtggatacggatccgaa	pNH-TrxT linearization
Vector.REV	ggattggaagtacaagttctcggtac	pNH-TrxT linearization
RPSA Fragment.FOR	ttgtacttccaatccATGTCCGGAGCCCTTGATG	RPSA(uS2) PCR amplification from OHU24758D
RPSA Fragment.REV	tatccacctttactgTCAAGACCAGTCAGTGGTTGCTCC	RPSA(uS2) PCR amplification from OHU24758D
RPS2 Fragment.FOR	ttgtacttccaatccATGGCGGATGACGCCG	RPS2(uS5) PCR amplification from OHU03177D
RPS2 Fragment.REV	tatccacctttactgTCATGTTGTAGCCACAGCTGGAG	RPS2(uS5) PCR amplification from OHU03177D
RPS3 Fragment.FOR	ttgtacttccaatccATGGCAGTGCAAATATCCAAGAAGAG	RPS3(uS3) PCR amplification from OHU16953D
RPS3 Fragment.REV	tatccacctttactgTCATGCTGTGGGGACTGGCT	RPS3(uS3) PCR amplification from OHU16953D
RPS7 Fragment.FOR	ttgtacttccaatccATGTTCAGTTCGAGCGCCAAG	RPS7(eS7) PCR amplification from OHU18854D
RPS7 Fragment.REV	tatccacctttactgTCACAATTGAAACTCTGGGAATTCAAAATTAACATCC	RPS7(eS7) PCR amplification from OHU18854D
RPS8 Fragment.FOR	ttgtacttccaatccATGGGCATCTCTCGGGACA	RPS8(eS8) PCR amplification from OHU31095D
RPS8 Fragment.REV	tatccacctttactgTCATTTGCCTTTGCGGGCCT	RPS8(eS8) PCR amplification from OHU31095D
RPS12 Fragment.FOR	ttgtacttccaatccATGGCCGAGGAAGGCATTG	RPS12(eS12) PCR amplification from OHu30883
RPS12 Fragment.REV	tatccacctttactgTCATTTCTTGCATTTGAAATACTCTTCAATGACATCC	RPS12(eS12) PCR amplification from OHu30883
RPS13 Fragment.FOR	ttgtacttccaatccATGGGTCGCATGCATGC	RPS13(uS15) PCR amplification from OHU13042D
RPS13 Fragment.REV	tatccacctttactgTCATGCGACCAGGGCAGAGG	RPS13(uS15) PCR amplification from OHU13042D
RPS14 Fragment.FOR	ttgtacttccaatccATGGCACCTCGAAAGGGGA	RPS14(uS11) PCR amplification from OHU10378D
RPS14 Fragment.REV	tatccacctttactgTCACAGACGGCGACCACG	RPS14(uS11) PCR amplification from OHU10378D

Table 3. Plasmids used in this study*

**Table:** 

Plasmid	Description	Source
pNH-TrxT	Empty backbone for ligase-independent cloning and bacterial expression of N-terminally tagged 6xHis-Thioredoxin -TEV fusion proteins	Gift from Opher Gileadi (Addgene plasmid # 26106)
pNH-TrxT-RPSA(uS2)	Recombinant plasmid for inducible bacterial expression of human RPSA(uS2) with N-terminal 6xHis-Thioredoxin tag	This study
pNH-TrxT-RPS2(uS5)	Recombinant plasmid for inducible bacterial expression of human RPS2(uS5) with N-terminal 6xHis-Thioredoxin tag	This study
pNH-TrxT-RPS3(uS3)	Recombinant plasmid for inducible bacterial expression of human RPS3(uS3) with N-terminal 6xHis-Thioredoxin tag	This study
pNH-TrxT-RPS7(eS7)	Recombinant plasmid for inducible bacterial expression of human RPS7(eS7) with N-terminal 6xHis-Thioredoxin tag	This study
pNH-TrxT-RPS8(eS8)	Recombinant plasmid for inducible bacterial expression of human RPS8(eS8) with N-terminal 6xHis-Thioredoxin tag	This study
pNH-TrxT-RPS12(eS12)	Recombinant plasmid for inducible bacterial expression of human RPS12(eS12) with N-terminal 6xHis-Thioredoxin tag	This study
pNH-TrxT-RPS13(uS15)	Recombinant plasmid for inducible bacterial expression of human RPS13(uS15) with N-terminal 6xHis-Thioredoxin tag	This study
pNH-TrxT-RPS14(uS11)	Recombinant plasmid for inducible bacterial expression of human RPS14(uS11) with N-terminal 6xHis-Thioredoxin tag	This study

*Plasmids constructed for this study have been deposited at Addgene
